# Differentinating between non-transfusion dependant β-thalassemia and iron deficinecy anemia in children using ROC and logistic regression analysis: two novel discrimination indices designed for pediatric patients

**DOI:** 10.3389/fped.2023.1258054

**Published:** 2024-01-16

**Authors:** Daniel Turudic, Jerko Vucak, Svetlana Kocheva, Danko Milosevic, Ernest Bilic

**Affiliations:** ^1^ Department of Pediatric Hematology and Oncology, University Hospital Centre Zagreb, Zagreb, Croatia; ^2^ Primary Health Care Pediatrician, Šibenik, Croatia; ^3^ University Clinic for Children’s Disease, Medical Faculty, Ss. Cyril and Methodius University in Skopje, Skopje, North Macedonia; ^4^ Macedonian Academy of Sciences and Arts, Skopje, Republic of Macedonia; ^5^ Croatian Academy of Medical Sciences, Zagreb, Croatia; ^6^ School of Medicine, University of Zagreb, Zagreb, Croatia

**Keywords:** β-thalassemia, iron deficiency anemia, discrimination index, classification, generalized linear models (GLM), children

## Abstract

**Introduction:**

This cross-sectional study enrolled a group of 271 children with microcytic anemia in order to test the performance of 41 single and 2 composite formulas andindices in distinguishing between β-thalassemia (β-thal) and iron deficiency anemia (IDA) in the pediatric population.

**Methods:**

Optimal pediatric cut-off values from the previously published formulas and indices were generated using ROC analysis. Logistic regression in R using generalized linear models (GLM) generated two new indices.

**Results:**

Formulas and indices with optimal cut-offvalues in children with accuracy ≥90% were (in descending order): Matos & Carvalho index, MDHL(Telmissani) formula, England and Fraser formula, Pornprasert index, Sirachainan index, Telmissani (MCHD) formula, CRUISE index, Hameed index, Sargolzaie formula and Zaghloul II index. The CroThalDD-LM1 index has an accuracy of 93.36% (AUC 0.986, 95% CI 0.975–0.997), while the second CroThalDD-LM2 index utilizes absolute reticulocyte count alongside CBC variables, with an accuracy of 96.77% (AUC 0.985, 95% CI 0.988–0.999).

**Discussion and conclusion:**

We recommend using aforementioned formulas and indices with corrected cut-off values and accuracy >90% alongside two new proposed indices. A comparison of both native and these new indices is encouraged. These are the first discrimination indices generated and designed precisely for the pediatric population, which includes preschool children.

## 1 Introduction

The discrimination between β-thalassemia (β-thal) and iron deficiency anemia (IDA) is of great socioeconomic importance. A recent multicenter study reports a 32.4% prevalence of IDA among infants in northwestern and 12% in children one year and older in central Croatia (1). Accurate and early diagnosis reduces costly and unnecessary laboratory testing. Furthermore, inappropriate empirical supplementary iron treatment, could be detrimental to the child's health. Many developing countries have limited resources for the genetic testing of β-thal, but have standard laboratory techniques for its detection. Since hemoglobin electrophoresis is not always readily available in developing countries, such discriminating formulas and indices can reduce their economic burden. Using accurate formula or index also provides an early screening with a high probability of β-thal or IDA in children. In this article, our goal was to determine the accuracy of the existing single and composite formulasand indices in distinguishing between these two diseases and, if possible, generate a new one(s). Previous adult indices and formulas with their original cut-off values have unsatisfactory diagnostic accuracy in distinguishing β-thal from IDA in children. Since children are the most vulnerable age group, especially in developing countries, creating a pediatric index that includes school and preschool children is necessary. The importance of this topic is proven by the latest works (1, 2).

## 2 Methods

To this end, we extensively searched three available databases (Pubmed, Scopus, Web of Science) to find all available published formulasand indices distinguishing β-thal from IDA. We tested the accuracy of forty-one single formulas andindices and two composite indices. The accuracy of these aforementioned formulasand indices has not been tested in children thus far. The following are shown in alphabetical order:

Alparslan [log10 (MCH × MCHC × RDW/RBC)], Bessman (RDW), Bordbar (80−MCV│ × │27−MCH), CRUISE (MCHC + 0.603 × RBC + 0.523 × RDW), Das Gupta (1.89 × RBC-0.33 × RDW-3.28), Ehsani (MCV−(10 × RBC), England & Fraser [MCV−RBC−(5 × Hb)−3.4], Green & King (MCV × MCV × RDW/Hb × 100), Hameed (MCV2 × RDW/(RBC2 × Hb), Hisham (Hb × MCV × RDW/RBC2), Huber-Herklotz (MCH × RDW × 0.1/RBC) + RDW), Index26, Jayabose (RDWI) (MCV × RDW/RBC), Janel (11T), Kandhro (RBC/HCT + 0.5 × RDW), Kandhro-2 (RDW × 5)/RBC), Keikhaei [Hb × RDW × 100/(RBC)2 × MCHC], Kerman I (MCV × MCH/RBC), Kerman II (MCV × MCH × 10/RBC × MCHC), Matos-Carvalho (1.91 × RBC) + (0.44 × MCHC), Mentzer (MCV/RBC), Merdin-1(RDW × RBC/MCV), Merdin-2 (RDW × RBC × Hb/MCV), Pornprasert (MCHC), Ravanbakhsh F1 (MCV/HCT), Ravanbakhsh F2 (RDW−3 × RBC), Ravanbakhsh F3 (MCV × RDW−100 RBC), Ravanbakhsh F4 (MCV × Hb/RDW × RBC), RBC (RBC), Ricerca (RDW/RBC), Sargolzaie (125.6 + (44.3 × RBC)−(20.9 × Hb)−(2.5 × MCV) + (20.3 × MCH)−12.18 × MCHC), Seghal (MCV2 /RBC), Shine-Lal (MCV × MCV × MCH/100), Sirachainan (1.5Hb-0.05 ×  MCV), Sirdah (MCV−RBC−(3 × Hb), Srivastava (MCH/RBC), SVM (1.45 × (MCV−82.8)/10.28 + 0.66 × (MCH−27.0)/3.9 + 0.98), Telmissani MCHD (MCH/MCV), Telmissani MDHL (MCH × RBC/MCV), Thal-index (Nishad) (0.615 × MCV) + (0.51 × MCH) + (0.446 × RDW), Wongprachum (MCV × RDW/RBC)−10Hb), Zaghloul I (Hb + Hct + RBC), Zaghloul II (Hb + Hct + RBC−RDW).

### 2.1 Inclusion and exclusion study criteria

• Inclusion criteria:
  ○ A complete blood count (CBC) analysis of children with microcytic anemia according to CALIPER hematology reference standards (3).
  ○ Age of children included ranged from 6 months to 18 years.
  ○ All patients with β-thal were non-transfusion dependant (NTDT) homozygous or compound heterozygous beta + thalassemia or heterozygous beta^0+^ thalassemia variants.
• Exclusion criteria:
  ○ Children under 6 months of age with low serum iron levels,
  ○ Children with iron supplementation therapy prior to inclusion into the study
  ○ Children with β-thal and low iron levels which could reduce the HbA2 level
  ○ Children receving recent blood transfusions
  ○ Children with other diseases causing microcytic hypochromic anemia (inflammatory, malignant, metabolic, cardiac, nephrological, or gastroenterological diseases, children with chronic illnesses, surgical procedures in children immediately before taking a blood sample for analysis),

CBC and reticulocyte analysis were performed on Sysmex XN-3000 automated blood analyzer for the values of red blood cell count (RBC), hemoglobin (Hb), hematocrit (Hct), mean corpuscular volume (MCV), mean corpuscular hemoglobin (MCH), mean corpuscular hemoglobin concentration (MCHC), red cell distribution width (RDW), reticulocytes (RTIC, *per mille* and absolute 10^12 ^/L). Peripheral blood samples were obtained by standard venipuncture. The osmotic fragility test assessed red cell osmotic fragility. Hb A, Hb A2, Hb F, and Hb variants were detected using an automated capillary electrophoresis system alongside high-performance liquid chromatography (HPLC) and isoelectric focusing (IEF) for confirmation. Hb A2 values >4% or hemoglobin HbF > 5% indicated β-thal carriers, while HbA values >4% with HbF up to 50% indicated NTDT β-thal. These complementary methods were used simultaneously in the routine laboratory work-up of every sample to ensure that no methodologic or clerical errors were made. IEF and capillary electrophoresis avoided the insufficient distinguishing of Hb Lepore vs. Hb A2 by HPLC due to overlapping retention times. Using manual IEF, we ensured the sharpness of the bands on the gel. The main disadvantage of capillary electrophoresis is the poor separation of Hb S vs. Hb D. To avoid the disadvantages of each method, we decided that all three methods should give the same positive results for β-thal. Iron deficiency anemia was diagnosed by measuring serum iron (Fe), ferritin (FER) [Ferritin ELISA Kit, Demeditec Diagnostics, GmbH], unsaturated iron binding capacity (UIBC), and total iron binding capacity (TIBC). Serum iron levels <4 μmol/L and serum ferritin levels <15 ng/dl indicate IDA (4). Since IDA can reduce HbA2 levels, hemoglobin variant analysis was repeated after IDA was corrected (5). DNA analysis was performed if protein-based methods revealed an unknown variant or ambiguous result. Gene-specific PCR analysis of pediatric Croatian patients was achieved by Q5 High-Fidelity PCR Kit (New England Biolabs) according to the manufacturer's instructions. Direct DNA sequencing of the amplified PCR products was done at Macrogene Europe. DNA analysis revealed the following hemoglobin subunit β-gene mutations: IVS-I-2 (T->C) (HBB:c.92 + 2T>C); 5′UTR; + 20 (C>T) (HBB:c.-31C>T); IVS-II-745 (G->C) (HBB:c.316-106C>G); IVS-I-110 (G->A)beta + (HBB:c.93-21G>A); IVS-I-1(G->A) (HBB:c.92 + 1G>A); IVS-II-666 C>T(HBB:c.316-185C>T); IVS-II-16 G>C (HBB:c.315 + 16G>C); IVS-II-666 C>T (HBB:c.316-185C>T); HBB:c.9T>C; HBB:c.315 + 74T>G; c.316-135het_dupT; c.316-133A>G; c.93-54G>A; c.316-68_316-67het_insCGG; c.316-342delA; c.316-312delT; c.316-209delT. The following mutations in the independent test sample were determined International Reference Laboratory for Haemoglobinopathies, Research Centre for Genetic Engineering and Biotechnology (RCGEB) “Georgi D. Efremov,” Skopje, Republic of Macedonia. Genomic DNA was isolated following a standard phenol extraction/ethanol precipitation protocol. DNA analysis for β-globin gene mutations was performed via ABI PRISM SNaPshot Multiplex Kit (Life Technologies) and ABI PRISM™ Big Dye Terminator v.1.1 Kit (Life Technologies, Carlsbad, CA, USA) following manufacturer's instructions (6). The following hemoglobin subunit β-gene mutations were found: IVS-I-I, IVS-I-110 G>A, IVS-I-6, c.118C>T (Cd39 C>T), c.20delA (Cd6-A), c.118C>T (Cd39 C>T), c.316-2A>G (IVS-II-897A>G), IVS-I-110 G>A, c.250delG (Cd82/83-G) ааа(anti 3.7) triplication, c.92 + 1G>A, c.118 C>T, g.34164_37967del3804 [a(-3.7)/aa], g.70676G>T (Cd27 G>T, Hb Knossos), c.93-21G>A (IVS-I-110 G>A), g.63489del [Cd59(-A)], g.70796G>A (IVS-I-110 G>A).

Informed parental consent was obtained in accordance with the Declaration of Helsinki.

### 2.2 Statistical analysis

The data analyzed were summarized as numbers and percentages. Quantitative data were summarized as the arithmetic mean and standard deviation. Depending on the data distribution, the data was analyzed using either parametric or nonparametric tests. Values of sensitivity, specificity, positive predictive value, negative predictive value, accuracy, Youden index, and area under the curve (AUC with 95% confidence limits) were used to assess the performance of individual formulasand indices. All applied tests were two-way; and *p* values ≤ 0.05 were considered statistically significant. The AIC (Akaike's Information Criterion) information criterion was used to select variables in the logistic regression model (7). Statistical analysis was performed in MedCalc ver. 19.07, StatSoft Statistica 12.5, and GraphPad Prism 8.4.3.686 (8, 9). Logistic regression analysis was performed in R version 4.1.3 (10). The optimal cut-off value for each formulaand index was based on the value of the area under the ROC curve as the value closest to the value of the area under the ROC curve in which the difference between the sensitivity and specificity was minimal (11).

## 3 Results

A total of 271 children of both genders were enrolled in the study, with 150 diagnosed with β-thal and 121 with IDA. All children with β-thal had non-transfusion dependant (NTDT) β-thal (β^+^/β^+^, β^0^/β^+^) or β-thal trait (β/β^0^, β/β^+^). There was no significant male-to-female ratio difference between groups (*χ*^2^−test *p* = 0.142). Children with IDA were younger than children with β-thal (median age 18 months for IDA vs. 74 months for β-thal). β-thal children had higher values of RBC, Hb, HCT, MCH, MCHC, and reticulocyte count (RTIC × 10^12^/L) than children with IDA. In contrast, children with IDA had higher RDW and MCV values than those with β-thal (*p* < 0.01). Values of MCV and RTIC (*per mille*) showed no differences between groups (*p* > 0.05). The results are shown in Table 1.

**Table 1:** Descriptive statistics comparing age, RBC, Hb, HCT, MCV, MCH, MCHC, RDW, RTIC (*per mille*), and RTIC (×10^12 ^/L) in children with β-thal and IDA (Mann–Whitney *U* test).

Variables	IDA (*n* = 121) M/F ratio (*n* = 1:1.2)	β-thal (*n* = 150) M/F ratio (*n* = 1:1.27)	*p*-value
	Arithmetic mean (standard deviation)	Arithmetic mean (standard deviation)	
Age (months)	51.44 (±63.74)	84.62 (±65.78)	*P* < 0.01
RBC (×10^12^)	4.78 (±0.68)	5.55(±0.59)	*P* < 0.01
Hb (g/L)	87.59 (±13.45)	111.09 (±12.86)	*P* < 0.01
HCT (%)	29.10 (±3.34)	34.09 (±3.63)	*P* < 0.01
MCV (fL)	62.02 (±7.89)	61.77 (±6.67)	*P* = 0.85
MCH (fL)	18.61 (±3.48)	20.17 (±2.73)	*P* < 0.01
MCHC	298.38 (±21.52)	325.83 (±11.76)	*P* < 0.01
RDW	18.75 (±2.99)	17.76 (±2.79)	*P* < 0.01
RTIC (per mile)	11.9 (±6.61)	11.07 (±4.79)	*P* = 0.90
RTIC (×10^12^/L)	57.04 (±26.63)	60.21 (±26.87)	*P* < 0.01
Iron (μmol/L)	3.73 (±1.53)	16.93 (±4.33)	*P* < 0.01
Ferritin (ug/L)	3.30 (±1.52)	51.53 (±34.24)	*P* < 0.01
TIBC (μmol/L)	85.07 (±9.3)	55.87 (±11.04)	*P* < 0.01
UIBC (μmol/L)	81.2 (±9.82)	38.93 (±12.09)	*P* < 0.01

Values *p* ≤ 0.05 are highlighted as statistically significant.

The performance of formulas and indices was tested with already published and new optimal cut-off values generated by ROC analysis.

Two formulas/indices with accuracy >80% using published cut-offs were (in descending order): Matos-Carvalho index and England and Fraser (Supplementary Table S1). The most accurate previously published index was the Matos-Carvalho index, both with with its original cut-off value for adults as well as an optimal cut-off value for children. With the previously published cut-off value, this index better distinguishes children with IDA than children with β-thal. With an optimal cut-off value, the accuracy of the Matos-Carvalho index in distinguishing β-thal increased (sensitivity 89.94%, specificity 93.75%). Therefore, the overall proportion of total misclassified patients is reduced and the index has better sensitivity and specificity.

The accuracy of many formulas and indices improved with the new optimal cut off values. Diagnostic accuracy of 10 indices, previously below 80%, increased with new optimal cut-off values above 80%. The accuracy of Pornprasert and RBC indices increased by more than >50% in comparison with published cut-off values. (Supplementary Table S2; Figure S1). Formulas and indices with optimal cut-off and accuracy between <90% and ≥80% are (in descending order): Telmissani (MDHL) formula, England and Fraser formula, Pornprasert (MCHC) index, Sirachainan index, Telmissani (MCHD) formula,CRUISE index, Hameed index,Sargolzaie formula, and Zaghloul II index. They became thereafter acceptable for distinguishing β-thal from IDA (Supplementary Table S2).

As we were not fully satisfied with the overall performance of the aforementined formulas and indices (primarily with sensitivity), we tried to improve diagnostic accuracy by creating a new model(s) suited for children. We applied binary classification using logistic regression (R for Windows 4.1.3). From a series of models generated using R.4.1.3, the most optimal ones were selected using AIC by combined addition and subtraction of variables. The variables used in the CroThalDD-LM1 model are MCH, MCV, and Hb. The model has the following calculation formula:

f(x)=exp⁡(y)/[1+exp⁡(y)]

where exp(y) = ey, y = β0 + β1 × 1 + β2 × 2 + β3 × 3. ×1 = MCH, ×2 = MCV, and ×3 = Hb. β0, β1, β2, β3 are coefficients of the logistic regression model (CroThalDD-LM1 index) shown in Table 2. The number calculated by CroThalDD-LM1 is between 0 and 1. The cut-off value is 0.5, and a number closer to 0 indicates the diagnosis of IDA, while a number closer to 1 indicates β-thal. Three variables built a model more accurate than any previous formulas/indices (composite indices included) with a sensitivity of 94.87%, specificity of 93.51%, accuracy of 94.10%, and AUC of 0.986 (95% CI 0.975–0.997) (Table 2).

**Table 2:** Variables and coefficients of the microcytic anemia via logistic regression model.

CrotThalDD-LM1					Confusion matrix for CroThalDD-1 model					
Coefficients	Estimate	SE	Z value	Pr(>|z|)		Baseline		Predicted		
(Intercept)	6.7405	3.8561	1.748	0.0805		Total	Total (%)	IDA	β-thal	Correct (%)
Hb	0.2392	0.0449	5.327	<0.0001	β-thal	150	55.4	6	144	94.87
MCH	2.6589	0.5101	5.212	<0.0001	IDA	121	44.6	111	10	93.51
MCV	−1.3346	0.2227	−5.992	<0.0001	Total: 271	94.10				
Akaike information criterion for CroThalDD-LM1 model 87.5546, bayesian information criterion for CroThalDD-LM1 101.9630										
CrotThalDD-LM2					Confusion matrix for CroThalDD-2 model					
Coefficients	Estimate	SE	Z value	Pr(>|z|)		Baseline		Predicted		
(Intercept)	−129.3687	24.2628	−5.332	<0.0001		Total	Total (%)	IDA	β-thal	Correct (%)
RBC	6.4260	1.2693	5.063	<0.0001	β-thal	124	57.1	4	120	95.74
MCHC	0.2945	0.0564	5.226	<0.0001	IDA	93	42.9	90	3	97.56
RTIC	0.0647	0.0202	3.207	0.00134	Total: 217	96.77				
Akaike information criterion for CroThalDD-LM2 model 50.2686, bayesian information criterion for CroThalDD-LM2 63.7697										

The 2nd formula generated via logistic regression uses the same variables as the Matos-Carvalho index (RBC and MCHC) with the addition of reticulocytes. The formula reads: exp(y) = ey, y = β0 + β1 × 1 + β2 × 2 + β3 × 3. ×1 = RBC, ×2 = MCHC, ×3 = RTIC (×10^12 ^/L), β0, β1, β2, β3 are coefficients of the logistic regression model (CroThalDD-LM2 index) shown in Table 3. Three variables built a model with a sensitivity of 95.74%, specificity of 97.56%, accuracy of 96.77%, and AUC of 0.985 (95% CI 0.988–0.999). The addition of RTIC (absolute count) increased the overall accuracy of the model.

**Table 3:** The CroThalDD-1 formula tested on an independent sample of β-thal from The Republic of North Macedonia.

Coefficients	Estimate	Confusion matrix for CroThalDD-1 model					
CroThalDDLM1			Baseline		Predicted		
(Intercept)	6.7405		Total	Total (%)	IDA	β-thal	Correct (%)
Hb	0.2392	β-thal	33	48.53	3	30	90.91
MCH	2.6589	IDA	35	51.47	35	3	91.43
MCV	−1.3346	Total: 68					91.18

CroThalDD-2 formula could not be tested due to a lack of reticulocytes.

To simplify the use of the model in everyday clinical practice, we present Microsoft Office Excel and LibreOffice Calc spreadsheets, which use this model (presented in the Supplementary Excel spreadsheet). In this way, the user only needs to enter the values of MCH, MCV, Hb, MCHC RBC, and RTIC (×10^12 ^/L), and the spreadsheets automatically calculate the probability of belonging to each class.

The practitioner can also calculate the sum of the values obtained by the simplified formula y = β0 + β1 × 1 + β2 × 2 + β3 × 3 without additional calculation with the exponential equation.

The simplified CroThalDD-LM1 index calculation formula would be:

y=6.7405+0.2392×Hb+2.6589×MCH−1.3346×MCV

The simplified CroThalDD-LM2 index calculation formula would be:

y=−129.3687+6.4260×RBC+0.2945×MCHC+0.0647×RTIC(abs.count)

In this case, the cut-off value has changed to 0. Values below 0 belong to IDA and above 0 to β-thal.

**Figure 1:**
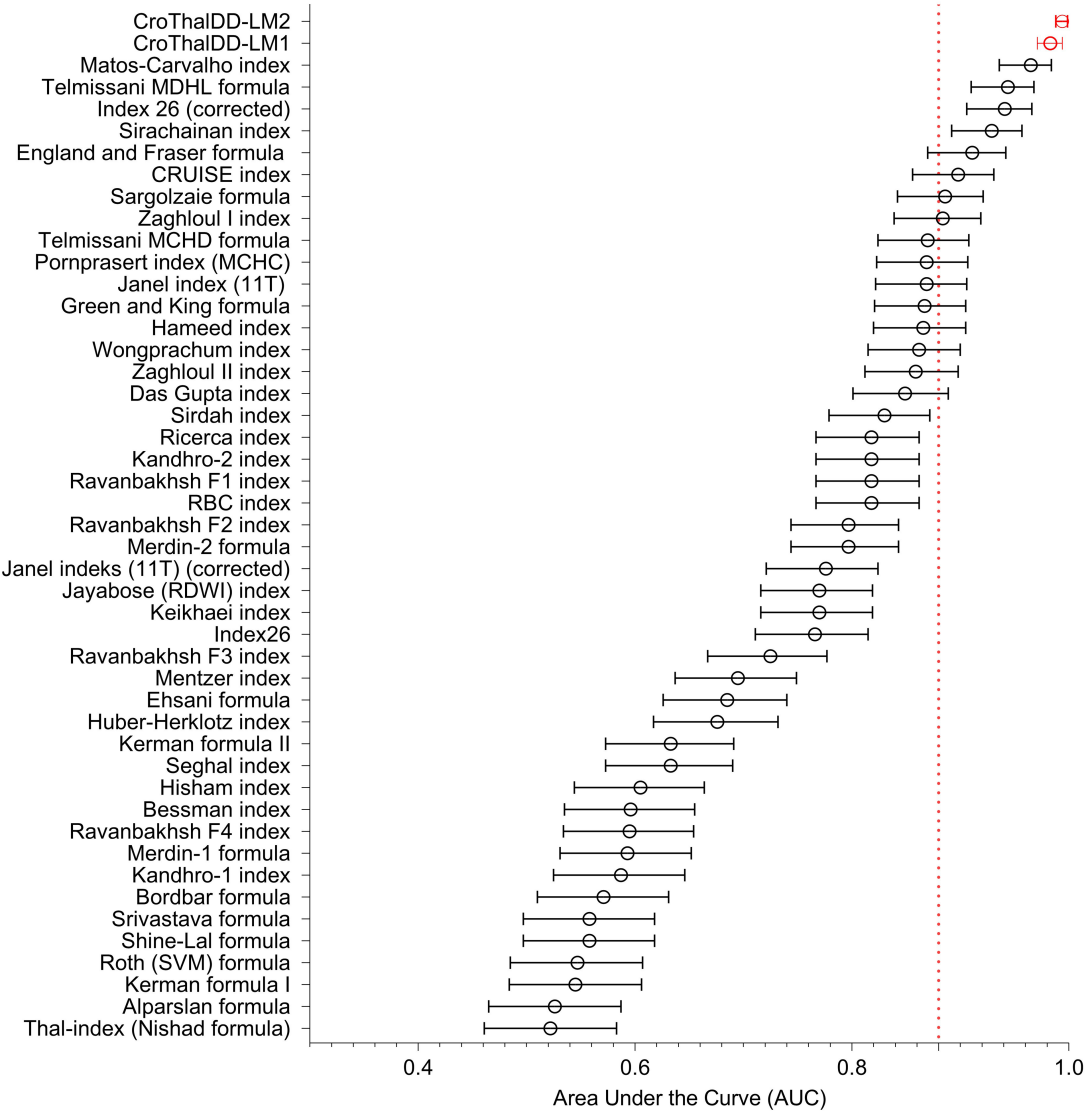
A summary of AUC values (with CI) of 41 formulas/indices, 2 composite indices and novel CroTHalDD-LM1 and CroThalDD-LM2 indices. The ten best indices have accuracy above 88% (marked by the dotted line on the x-axis).

A summary of AUC values (with CI) of 41 formulas/indices, 2 composite indices, and novel CroThalDD-LM1 and CroThalDD-LM2 indices are shown in Figure 1. The ten best indices have accuracy above 88% (marked by the dotted line on the x-axis).

The CroThalDD-1 index was additionally tested on a different population of children from the Republic of North Macedonia unrelated to the Croatian population. These populations are genetically different (12). A slight decrease in sensitivity and specificity was noticed but with still high accuracy. The results are shown in Table 3. Due to the lack of RTIC in the North Macedonian blood samples, the CroThalDD-2 index was not tested.

## 4 Discussion

Since the appearance of the Mentzer index several decades ago, attempts have been made to find a universal and reliable formula or index to distinguish β-thal from IDA. Sensitivity, specificity, PPV, NPV, accuracy, AUC, and Youden index were analyzed in most discrimination formulas and indices, but their performance differed worldwide. This is especially true for children, for whom the diagnostic accuracy of previously published discrimination formulasand indices showed lower performancethan in adults. Differential diagnosis is especially challenging when both IDA and β-thal are present simultaneously. Elevated erythropoietin levels due to sustanined anemia in β-thal stimulate the release of erythroferrone through erythroblasts (13, 14). This increase in erythroferrone, in turn, supresses hepcidine expression. The resulting increased intestinal iron absorption and with the release of recycled iron from the reticuloendothelial system lead to portal and hepatocyte iron loading, and free circulating iron causes organ damage. Therefore, additional unnecessary long-term iron supplementation leads to iron accumulation and with potential deleterious side effects.

IDA in children becomes clinically apparent at the age of 12 months, and the American Academy of Pediatrics recommends screening infants around 1 year of age due to reduced iron storage. NTDT β-thal typically presents between the ages of 2–4 years. Our results correspond to these age groups (15, 16). A higher RDW value of IDA indicates more significant heterogeneity (anisocytosis) of β-thal, with higher or average RDW values (17). In the case of progressing or long-standing IDA, RBC count and MCV may be decreased due to fewer and smaller erythrocytes being produced in IDA due to depletion of iron stores *vs.* β-thal.

Most formulasand indices have been generated and tested in adult populations with various discriminating performances in children. Indices with ≥90% accuracy were rated as the most accurate and recommended in a clinical setting for distinguishing β-thal from IDA in Croatian children. Indices with an accuracy between ≥80% and <90% can help to differentiate between these two diseases. Indices with accuracy <80% were not recommended due to many formulasand indices with better performances.

The Matos-Carvalho index has proven to be the most accurate of all formulas and indices in Croatian children, even with the previously published cut-off value described in the literature (18). CBC values on which the initial index was created differ from those in Croatian children, as one depends on the genetic background of patients with β-thal. Due to Croatia's association with the Mediterranean population, high accuracy of the Matos-Carvalho index is expected. A similar performance of the Matos-Carvalho index was observed in the adult Egyptian population (19). This index has not been previously tested in children.

The second-best index with an optimal cut-off value was Telmissani (MDHL) index. The performance rating of our study corresponds to a study of adult patients conducted in Saudi Arabia (21). Both Telmissani indices (MDHL and MCHD) with optimal cut-off values can be recommended for routine tesing in children. They have not been tested in children so far as well. With the published cut-off, the England and Fraser index had the second-best performance rating (22, 23). The original cut-off value of the index showed excellent sensitivity, NPV, and accuracy but insufficient specificity and PPV. After defining the optimal cut-off value and performance improvement, the accuracy of the index increased due to improved specificity and PPV, and it ranked fifth best in terms of performance. England and Fraser formula was previously tested in the population of children in Turkey and had a third-best-ranked performance (below RDWI and RBC indices) (24, 25). This index also proved third best in Chinese children (below RDWI and Green and King indices) (26).

The Pornprasert index uses only MCHC as a discrimination value (27). After defining the optimal cut-off value, its accuracy increased by 65% due to an increase in sensitivity and specificity. The index was created for Thai school children; therefore, a favorable result of the discriminatory performance of this index among Croatian children for the two diseases is expected. Since our study population encompasses toddlers and preschool children, an MCHC cut-off value >309 corresponds with the diagnosis of β-thal in the entire population of children. Thai and Croatian populations have different median ages for β-thal, so the difference in cut-off value is understandable. The Sirachainan index was also generated on a population of healthy school children, and excellent initial performance of this index was also expected (28). After defining the optimal cut-off value, its accuracy improved substantially by 13.92%, and the index proved valuable in distinguishing children with β-thal from children with IDA.

The last four indices which improved accuracy after defining optimal cut-off values >80% were CRUISE, Hameed, Sargolzaie, and Zaghloul II. After defining the optimal cut-off value, the CRUISE index outperformed the formulas/indices initially developed for the adult Iranian population (29). Hameed index with the new cut-off value showed balanced sensitivity and specificity with acceptable accuracy (19). After defining the optimal cut-off value, the Sargolzaie index also significantly increased its accuracy (30). Defining the cut-off values of the Zaghloul II index in children (regardless of gender), the index showed a significant improvement in accuracy mainly due to its increase in specificity and PPV (31). All of these indices have not been tested in children so far.

Using linear regression components embedded in the logit scale, logistic regression iteratively identifies the combination of variables with the most significant probability of discriminating between β-thal and IDA. Logistic regression analysis generated two new discriminations indices distinguishing children with β-from IDA in children with the highest sensitivity, specificity, and accuracy of all formulas/indices, as mentioned earlier.

The RTIC count (per *mille* and absolute reticulocyte number) is high in the case of active β-thal but also severe IDA (20) due to erythropoietin stimulation. A significanlty higher reticulocyte count was already observed in NTDT thalassemia patients, in comparison with IDA (32). Since the Matos-Carvalho index shows the best results in our children, we put to the test the performance of the variables of this index (RBC, MCHC) with the addition of RTIC. The result is the new CroThalDD-LM2 index. Adding RTIC usually improves the performance of some of the already published formulas/indices.

The results of our study are logistic model-generated indices, the first discrimination indices designed precisely for the pediatric population, including preschool children. To make the calculation with the exponential equation more accessible, we created Excel and Libre Office spreadsheets. Even though CroThalDD-LM2 has better accuracy, sensitivity, and CI, we suggest using both novel formulas/indices to see which has the advantage in practical application. Although these indices are derived from Croatian children database, we encourage their use in other children populations, even adults.

We tested all available formulas/indices of β-thal of native adult origin and adopted them for use in our children population. We hope that our results will help practitioners already accustomed to native formulas/indices to improve their reliability of β-thal diagnostics in children. All modified cut-offs of such native formulas/indices are listed in the Supplementary Files S1. A comparison between such native formulas/indices and our novel-generated indices is very welcome (33–35).

### 4.1 Limitations of the study

The indices using the average reticulocyte cell volume, the average reticulocyte hemoglobin content, % Micro-R, or mean platelet volume to separate β-thal from IDA were not analyzed as they are not routine in routine analysis in Croatia or North Macedonia (36, 37). We encountered only four children with α-thalassemia trait, and therefore, we cannot support the usability of these indicies in α-thalassemia trait. Further research is required.

## 5 Conclusions

All tested formulas/indices which are generated worldwide were tested and modified on the children population. The Matos-Carvalho index shows the best diagnostic performance for distinguishing β-thal from IDA in children, We recommend the use of formulas/indices with modified cut-offs with a performance of >90% accuracy. We generated two novel indices from the children in order to reflect the peculiarities in β-thal diagnostics in such populations, which were observed by previous authors by using formulas/indices generated from the adults. A comparison of both native and our indices is encouraged.

## Data Availability

The raw data supporting the conclusions of this article will be made available by the authors, without undue reservation.
