# Supplementary material for: Differentinating between non-transfusion dependant β-thalassemia and iron deficinecy anemia in children using ROC and logistic regression analysis: two novel discrimination indices designed for pediatric patients
Source: Front Pediatr. 2024 Jan 16;11:1258054. doi: 10.3389/fped.2023.1258054 (PMC10824984; doi:10.3389/fped.2023.1258054)

Supplementary Table 1. Diagnostic performances of 41 formulas/indices and 2 composite indices before optimal cut-off values for children by ROC analysis.

| **Name** | **Sensitivity** | **Specificity** | **PPV** | **NPV** | **Accuracy** | **Cut-off** |
| --- | --- | --- | --- | --- | --- | --- |
| Alparslan formula | N/A | N/A | N/A | N/A | N/A | <3.34 |
| Bessman index | 51.72 | 44.21 | 10.00 | 88.43 | 45.02 | <15 |
| Bordbar formula | 57.58 | 57.50 | 88.67 | 19.01 | 57.56 | >44.76 |
| CRUISE index | 72.28 | 94.20 | 97.33 | 53.72 | 77.86 | ≥42.63 |
| Das Gupta index | 72.88 | 77.66 | 86.00 | 60.33 | 74.54 | >0 |
| Ehsani formula | 66.33 | 73.33 | 86.67 | 45.45 | 68.27 | <15 |
| England & Fraser formula | 93.75 | 71.60 | 75.76 | 92.37 | 82.37 | ≤0 |
| Green & King formula | 85.71 | 68.42 | 68.00 | 85.95 | 76.01 | ≤65 |
| Hameed index | 90.36 | 68.36 | 72.82 | 88.32 | 79.01 | <220 |
| Hisham index | 57.14 | 68.42 | 96.00 | 10.74 | 57.93 | <67 |
| Huber-Herklotz index | 65.98 | 85.51 | 86.49 | 64.13 | 74.10 | <21 |
| Index26 | 82.81 | 69.23 | 70.67 | 81.82 | 75.65 | >16 |
| Janel index (11T) | 94.95 | 67.44 | 62.67 | 95.87 | 77.49 | >8 |
| Jayabose (RDWI) index | 74.83 | 66.41 | 71.33 | 70.25 | 70.85 | ≤220 |
| Kandhro-1 index | 59.09 | 45.85 | 26.00 | 77.69 | 49.08 | <8.2 |
| Kandhro-2 index | 81.90 | 64.52 | 63.33 | 82.64 | 71.96 | <16.8 |
| Keikhaei index | 80.00 | 65.75 | 66.67 | 79.34 | 72.32 | <21 |
| Kerman formula I | 59.64 | 64.58 | 88.67 | 25.62 | 60.52 | <300 |
| Kerman formula II | 64.58 | 71.23 | 86.00 | 42.98 | 66.79 | <85 |
| Matos Carvalho index | 94.34 | 85.21 | 87.72 | 93.08 | 90.03 | >2385 |
| Mentzer index | 70.09 | 85.21 | 87.72 | 65.41 | 76.12 | ≤13 |
| Merdin-1 formula | 63.03 | 66.67 | 88.67 | 30.36 | 63.74 | >1.27 |
| Merdin-2 formula | 76.28 | 73.04 | 79.33 | 69.42 | 74.91 | >14.7 |
| Pornprasert index (MCHC) | 17.65 | 21.89 | 12.00 | 30.58 | 20.30 | <31.0 |
| Ravanbakhsh F1 index | 72.57 | 76.04 | 84.67 | 60.33 | 73.80 | <2 |
| Ravanbakhsh F2 index | 84.91 | 63.64 | 60.00 | 86.78 | 71.96 | <1.5 |
| Ravanbakhsh F3 index | 73.38 | 63.64 | 68.00 | 69.42 | 68.63 | <600 |
| Ravanbakhsh F4 index | 60.24 | 88.97 | 90.91 | 55.00 | 70.39 | <10 |
| RBC index | 24.53 | 24.39 | 17.33 | 33.33 | 24.44 | <5.1 |
| Ricerca index | 59.67 | 82.14 | 96.67 | 19.01 | 61.99 | <4.4 |
| Roth (SVM) formula | 54.86 | 35.71 | 94.00 | 4.13 | 53.87 | <0 |
| Sargolzaie formula | 88.98 | 70.59 | 70.00 | 89.26 | 78.60 | <0.5 |
| Seghal index | 60.63 | 68.00 | 89.33 | 28.10 | 61.99 | <972 |
| Shine-Lal formula | 54.86 | 35.71 | 94.00 | 4.13 | 53.87 | <1530 |
| Sirachainan index | 73.89 | 68.36 | 72.82 | 69.54 | 71.32 | >14 |
| Sirdah index | 75.61 | 75.70 | 82.67 | 66.94 | 75.65 | ≤27 |
| Srivastava formula | 63.69 | 58.25 | 71.33 | 49.59 | 61.62 | ≤3.8 |
| Telmissani (MCHD) formula | 55.76 | 91.67 | 93.17 | 50.42 | 67.58 | <0.34 |
| Telmissani (MDHL) formula | 56.18 | 57.35 | 62.50 | 50.84 | 56.69 | <1.75 |
| Thal-index (Nishad formula) | 56.93 | 50.00 | 76.67 | 28.69 | 55.15 | <59 |
| Wongprachum index | 87.39 | 69.74 | 69.33 | 87.60 | 77.49 | <104 |
| Zaghloul I index | 92.45 | 53.67 | 32.67 | 96.69 | 61.25 | >52.5 |
| Zaghloul II index | 88.89 | 49.79 | 21.33 | 96.69 | 54.98 | >37,1 |

Supplementary Table 2. Diagnostic performances of 41 formulas/indices and 2 composite indices with optimal cut-offs.

| **Name** | **Sensitivity** | **Specificity** | **PPV** | **NPV** | **Accuracy** | **AUC** | **YI** | **Cut-off** |
| --- | --- | --- | --- | --- | --- | --- | --- | --- |
| Alparslan formula | 78.02 | 91.01 | 94.67 | 66.94 | 82.29 | 0.888 | 0.0866 | ≤4.416 |
| Bessman index | 61.86 | 61.04 | 80 | 38.84 | 61.62 | 0.596 | 0.1833 | ≤19.6 |
| Bordbar formula | 63.48 | 90.24 | 97.33 | 30.58 | 67.53 | 0.571 | 0.2791 | ≤252.84 |
| CRUISE index | 85.52 | 79.37 | 82.67 | 82.64 | 82.66 | 0.898 | 0.6614 | >43.97 |
| Das Gupta index | 92.86 | 65.9 | 60.67 | 94.21 | 75.65 | 0.849 | 0.5488 | >0.98 |
| Ehsani formula | 68.93 | 70.21 | 81.33 | 54.55 | 69.37 | 0.685 | 0.3655 | ≤12.5 |
| England & Fraser formula | 90.34 | 84.92 | 87.33 | 88.43 | 87.82 | 0.911 | 0.7576 | ≤2.9 |
| Green & King formula | 84.96 | 73.19 | 75.33 | 83.47 | 78.97 | 0.867 | 0.5947 | ≤69.28 |
| Hameed index | 81.65 | 81.42 | 86 | 76.03 | 81.55 | 0.866 | 0.6203 | ≤275.32 |
| Hisham index | 68.32 | 52.35 | 46 | 73.55 | 58.3 | 0.605 | 0.1955 | ≤36.16 |
| Huber-Herklotz index | 85.92 | 55.5 | 40.67 | 91.74 | 63.47 | 0.676 | 0.324 | ≤22.32 |
| Index26 | 81.62 | 71.11 | 74 | 79.34 | 76.38 | 0.819 | 0.5334 | >5 |
| Janel index (11T) | 80,27 | 75.33 | 75.33 | 93.39 | 83.39 | 0.825 | 0.825 | >6 |
| Jayabose (RDWI) index | 82.2 | 65.36 | 64.67 | 82.64 | 72.69 | 0.77 | 0.4731 | ≤204.62 |
| Kandhro-1 index | 61.54 | 60.53 | 80 | 38.02 | 61.25 | 0.587 | 0.1868 | ≤9.97 |
| Kandhro-2 index | 80.29 | 70.15 | 73.33 | 77.69 | 75.28 | 0.818 | 0.5169 | ≤17.38 |
| Keikhaei index | 82.05 | 64.94 | 64 | 82.64 | 72.32 | 0.77 | 0.4731 | ≤20.46 |
| Kerman formula I | 63.37 | 68.12 | 85.33 | 38.84 | 64.58 | 0.545 | 0.2484 | ≤272.18 |
| Kerman formula II | 68.05 | 65.69 | 76.67 | 55.37 | 67.16 | 0.633 | 0.3204 | ≤75.52 |
| Matos-Carvalho index | 89.94 | 93.75 | 95.33 | 86.78 | 91.51 | 0.965 | 0.8211 | >23.44 |
| Mentzer index | 76.88 | 72.41 | 78.14 | 70.95 | 74.92 | 0.695 | 0.378 | ≤11.68 |
| Merdin-1 formula | 66.48 | 65.26 | 78 | 51.24 | 66.05 | 0.593 | 0.2924 | >1.41 |
| Merdin-2 formula | 75.78 | 74.55 | 81.33 | 67.77 | 75.28 | 0.797 | 0.4993 | >14.49 |
| Pornprasert index (MCHC) | 79.44 | 94.17 | 95.33 | 75.33 | 85.33 | 0.869 | 0.6409 | >30.90 |
| Ravanbakhsh F1 index | 77.42 | 74.14 | 80 | 71.07 | 76.01 | 0.818 | 0.5174 | ≤1.94 |
| Ravanbakhsh F2 index | 92.05 | 62.3 | 54 | 94.21 | 71.96 | 0.797 | 0.4888 | ≤1.02 |
| Ravanbakhsh F3 index | 82.24 | 62.2 | 58.67 | 84.3 | 70.11 | 0.725 | 0.4363 | ≤534.21 |
| Ravanbakhsh F4 index | 67.65 | 82.09 | 92 | 45.45 | 71.22 | 0.595 | 0.3783 | >5.21 |
| RBC index | 77.22 | 75.22 | 81.33 | 70.25 | 76.38 | 0.818 | 0.5158 | >5.14 |
| Ricerca index | 80.29 | 70.15 | 73.33 | 77.69 | 75.28 | 0.818 | 0.5169 | ≤3.48 |
| Roth (SVM) formula | 62.55 | 61.45 | 78.67 | 42.15 | 62.36 | 0.547 | 0.261 | >-4,73 |
| Sargolzaie formula | 84.62 | 77.34 | 80.67 | 81.82 | 81.18 | 0.886 | 0.6315 | ≤3.42 |
| Seghal index | 68.26 | 65.38 | 76 | 56.2 | 67.16 | 0.633 | 0.322 | ≤751.68 |
| Shine-Lal formula | 62.66 | 89.47 | 97.33 | 28.1 | 66.42 | 0.558 | 0.2626 | >506.71 |
| Sirachainan index | 88.73 | 81.4 | 84 | 86.78 | 85.24 | 0.929 | 0.7078 | >11.93 |
| Sirdah index | 84.13 | 69.66 | 70.67 | 83.47 | 76.38 | 0.83 | 0.548 | ≤24.36 |
| Srivastava formula | 64.61 | 62.37 | 76.67 | 47.93 | 63.84 | 0.558 | 0.2527 | ≤3.92 |
| Telmissani (MCHD) formula | 79.33 | 91.3 | 94.67 | 69.42 | 83.39 | 0.87 | 0.6409 | >0.31 |
| Telmissani (MDHL) formula | 88.05 | 91.07 | 93.33 | 84.3 | 89.3 | 0.944 | 0.7763 | >1.57 |
| Thal-index (Nishad formula) | 60.17 | 72.5 | 92.67 | 23.97 | 61.99 | 0.522 | 0.1663 | >50.66 |
| Wongprachum index | 85.16 | 71.33 | 72.67 | 84.3 | 77.86 | 0.862 | 0.5763 | ≤111.70 |
| Zaghloul I index | 89.08 | 71.05 | 70.67 | 89.26 | 78.97 | 0.884 | 0.5992 | >48.21 |
| Zaghloul II index | 81.93 | 85.42 | 90.67 | 73.21 | 83.21 | 0.859 | 0.5926 | >26.43 |

Supplementary Figure 1. Difference in accuracy between original (O) and optimal (OA) cut-off values .


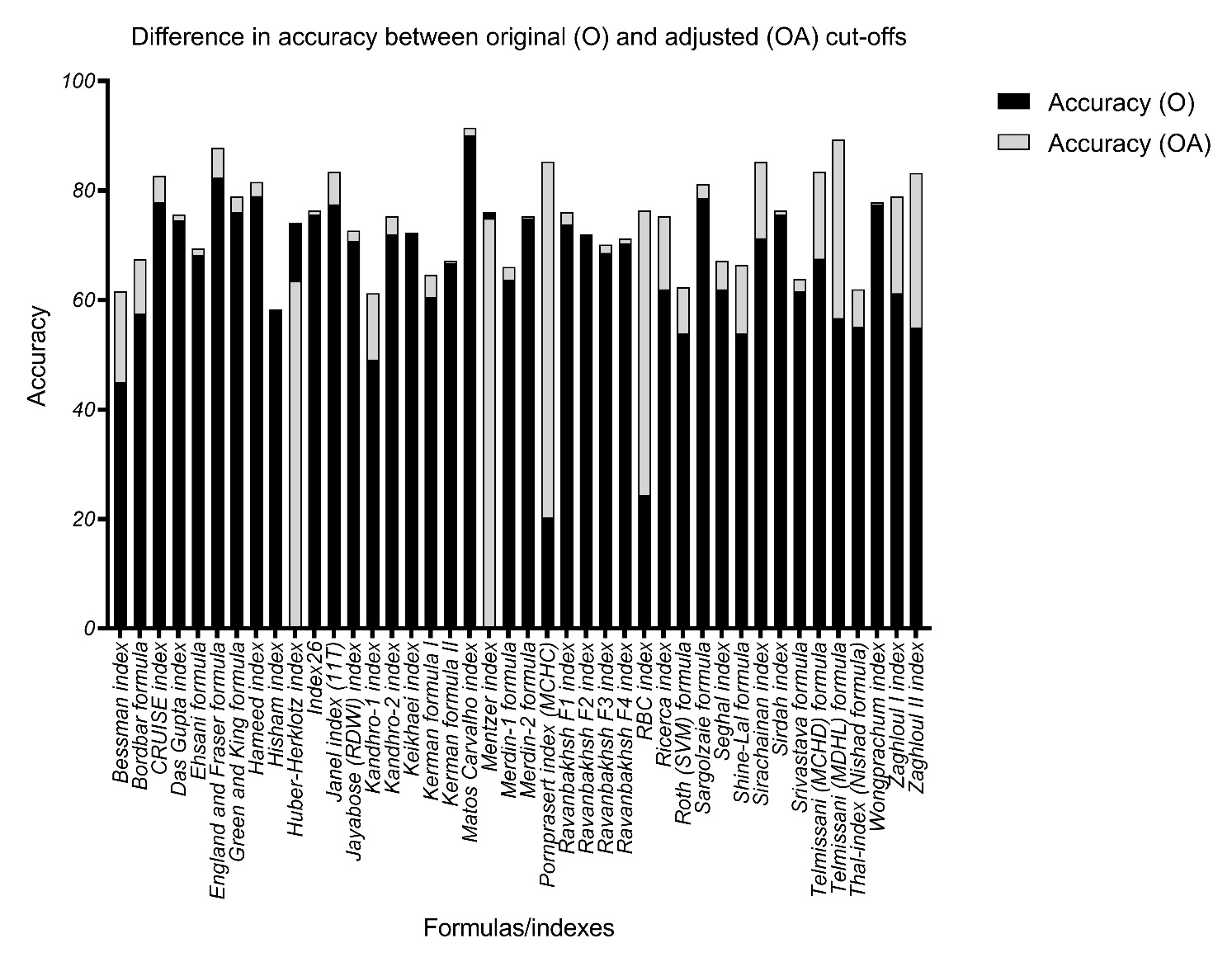

Supplement: Supplementary file 1 [file Table1.docx]
