# Supplementary figures and images for: Differentinating between non-transfusion dependant β-thalassemia and iron deficinecy anemia in children using ROC and logistic regression analysis: two novel discrimination indices designed for pediatric patients
Source: Front Pediatr. 2024 Jan 16;11:1258054. doi: 10.3389/fped.2023.1258054 (PMC10824984; doi:10.3389/fped.2023.1258054)

Supplementary Figure 1. Difference in accuracy between original (O) and optimal (OA) cut-off values .

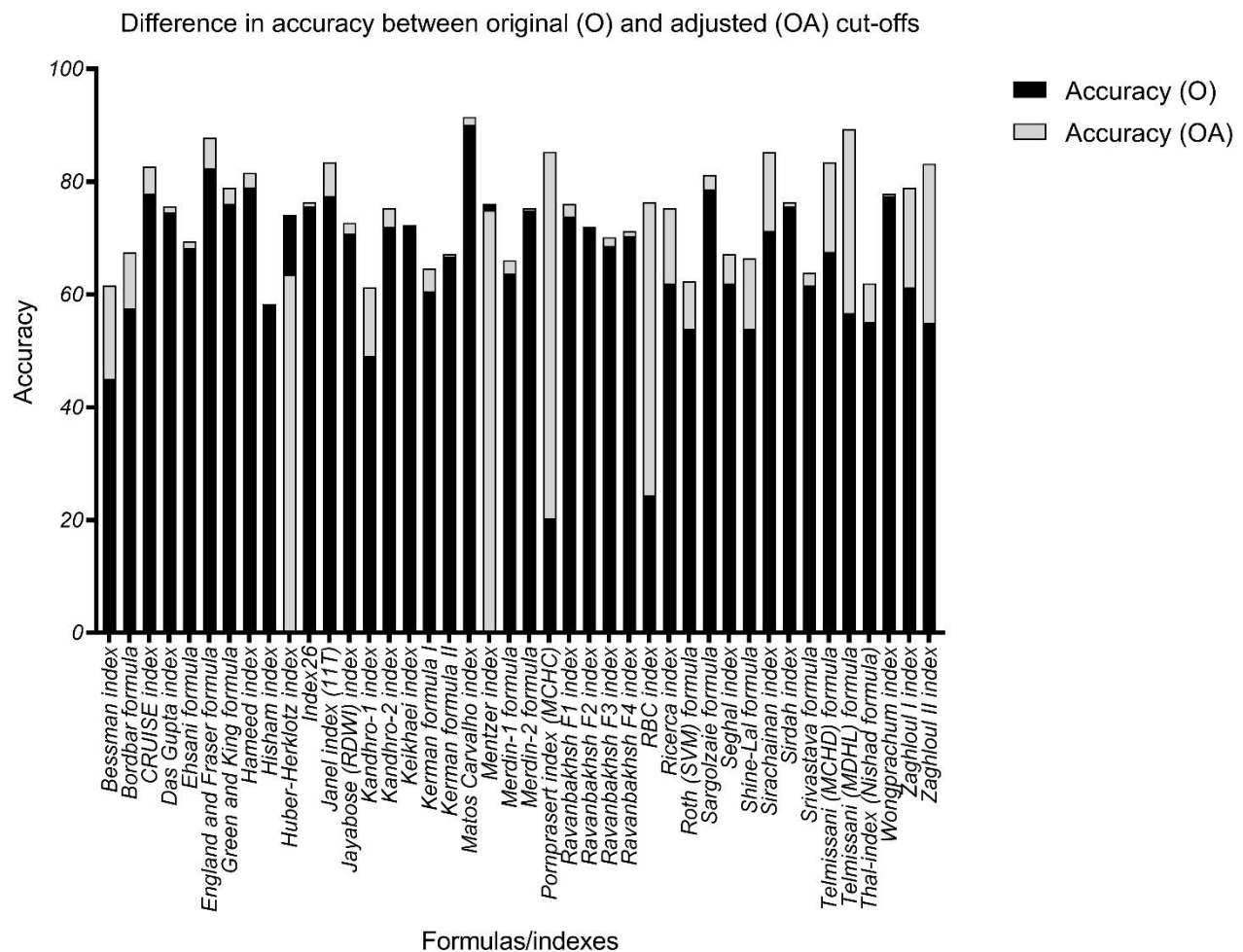

Supplement: Supplementary file 3 [file Image1.pdf]
